# Network Disconnection Syndrome in Unruptured Brain Arteriovenous Malformations: A Multimodal Connectome Study

**DOI:** 10.1002/cns.70819

**Published:** 2026-03-11

**Authors:** Xuchen Dong, Haojing Duan, Yingtao Liu, Zhiyuan Fan, Hongfei Zhang, Yingjun Liu, Zongze Li, Peixi Liu, Yuan Shi, Xiaolei Lin, Kai Quan, Wei Zhu

**Affiliations:** ^1^ Department of Neurosurgery, Huashan Hospital, Shanghai Medical College Fudan University Shanghai China; ^2^ National Center for Neurological Disorders Shanghai China; ^3^ Shanghai Key Laboratory of Brain Function and Restoration and Neural Regeneration Shanghai China; ^4^ Neurosurgical Institute Fudan University Shanghai China; ^5^ Shanghai Clinical Medical Center of Neurosurgery Shanghai China; ^6^ Institute of Science and Technology for Brain‐Inspired Intelligence Fudan University Shanghai China; ^7^ Department of Radiology, Huashan Hospital Fudan University Shanghai China; ^8^ Huashan Institute of Medicine, Huashan Hospital Fudan University Shanghai China; ^9^ School of Data Science Fudan University Shanghai China

**Keywords:** cerebral arteriovenous malformation, cognitive impairment, connectome, network diffusion model, structure–function coupling

## Abstract

**Background:**

Cognitive impairment is a critical yet poorly understood complication of brain arteriovenous malformations (bAVM). While traditionally attributed to hemodynamic “steal” or focal tissue destruction, it remains unclear whether cognitive variability correlates with gross structural pathology or broader network disruption.

**Methods:**

We recruited consecutive patients with unruptured bAVM at Huashan Hospital and matched healthy controls (2017–2020). We integrated lesion frequency mapping, surface‐based morphometry, and Network Diffusion Modeling (NDM) to dissect the structure–function relationship in a cohort of bAVM patients compared to matched controls. We systematically evaluated the predictive value of three distinct damage metrics—gross lesion load, cortical morphological remodeling, and white matter structural connectivity—on global and domain‐specific cognitive performance.

**Results:**

Patients with bAVM exhibited significantly reduced global efficiency in structural networks (*p* < 0.05), which was a stronger predictor of cognitive decline than lesion volume. In contrast, global functional network topology was largely preserved. Although local functional reorganization was observed, characterized by increased inter‐hemispheric synchronization, these adaptations did not prevent cognitive deficits. Notably, increased structure–function decoupling within high‐order association networks (including frontoparietal and ventral attention systems) was significantly associated with executive and memory impairment.

**Conclusions:**

Cognitive dysfunction in bAVM represents a disconnection syndrome caused by the disruption of long‐range white matter tracts rather than local cortical pathology. The dissociation between structural damage and functional compensation highlights the limitations of conventional volumetric assessment. Preoperative connectome mapping, particularly of white matter integrity, may improve risk stratification and functional preservation strategies.

## Graphical abstract

This study challenges the traditional reliance on lesion size, demonstrating that cognitive impairment in unruptured bAVMs is fundamentally driven by global white matter disconnection. We identified a critical “structure–function decoupling,” where compensatory reorganization fails once the underlying structural scaffold collapses. These findings highlight a physiological “ceiling of plasticity,” advocating for a paradigm shift toward tract‐sparing strategies in preoperative planning.

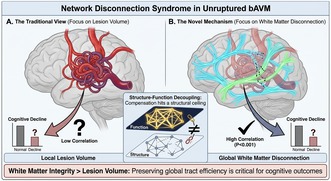

## 1 Introduction

The management of unruptured brain arteriovenous malformations (AVMs) remains controversial. While hemorrhage risk typically drives interventional decisions, the functional impact of these lesions is often underestimated. The pivotal ARUBA trial, which compared medical management alone vs. intervention, defined “poor outcome” narrowly as a modified Rankin Scale (mRS) score of ≥ 2. This definition perpetuated the misconception that unruptured AVMs are clinically “asymptomatic” in the absence of overt neurological deficits [1, 2, 3]. However, this exclusive focus on gross motor‐sensory function overlooks subtler, yet potentially disabling, cognitive impairments [4]. Recent evidence indicates that up to 71.4% of patients with unruptured AVMs exhibit specific deficits in memory, executive function, or attention—even with normal mRS scores [5]. These under‐recognized impairments significantly compromise quality of life, highlighting the limitations of current functional outcome measures.

Traditional understanding of AVM pathogenesis has centered on lesion‐centric mechanisms such as mass effect or regional hypoperfusion. This classical view assumes neurological deficits arise directly from structural damage at the lesion site and relies heavily on local hemodynamic signals, which may be unreliable in AVMs [6, 7, 8]. However, as AVMs represent developmental anomalies, emerging evidence challenges this static view. Studies on language lateralization, for instance, demonstrate that functional organization often deviates from canonical patterns due to chronic plasticity, suggesting that deficits correlate less with mere lesion location and more with the failure of developmental reorganization [9]. As described in brain tumor research, the developing brain exhibits considerable plasticity, with functional networks dynamically reorganizing to compensate for structural insults [4]. For AVMs, this suggests a transition in understanding—from viewing deficits as focal lesions to recognizing them as network disconnection syndromes. While the term “disconnection” historically encompassed overt sensorimotor deficits, we specifically posit a “Cognitive Disconnection Syndrome” in unruptured AVMs. In this phenotype, patients appear neurologically intact by conventional standards (mRS 0) due to robust sensorimotor pathway redundancy, yet suffer from “silent” network failures manifest strictly as high‐order executive and memory decline. In this model, the lesion interrupts inherent brain connectomes, partially mitigated by network redundancy [10, 11]. This “connectionist” framework better explains the heterogeneity of cognitive phenotypes, variable clinical presentations, and risks of intervention‐related iatrogenic disconnections [12, 13, 14].

To quantify these network alterations, computational approaches are critical. Network diffusion models (NDM), which simulate how pathological signals disrupt connectomic architectures, provide a quantitative method to map the topological basis of cognitive impairment [15]. By integrating structural connectivity data with clinical metrics, NDM can identify structural “epicenters” of network damage and predict the extent of functional compromise—even when masked by adaptive reorganization. Such methods may facilitate the translation of pathophysiological insights into risk stratification, guiding interventions to preserve network integrity [16].

In this study, we investigated the neural basis of cognitive impairment in unruptured AVMs using a connectomic approach. We hypothesized that AVM‐related cognitive deficits arise from chronic white matter disconnection, with patterns determined by connectome topology rather than lesion location alone. By applying NDM to structural imaging data, we aimed to elucidate the interaction between pathological spread and adaptive reorganization, ultimately informing network‐preserving surgical strategies.

## 2 Methods

### 2.1 Participants and Study Design

A consecutive cohort of patients with unruptured brain arteriovenous malformations (AVM) was recruited from Huashan Hospital, Fudan University, between January 2017 and June 2020. Inclusion required a radiologically confirmed diagnosis, completion of multimodal MRI scanning (high‐resolution T1, resting‐state fMRI, and DTI), and a comprehensive neuropsychological assessment covering global cognition, memory, language, executive function, and emotion within two weeks of enrollment. Patients with a history of hemorrhage, prior interventions, diffuse lesions, other neurological comorbidities, or significant motion artifacts were excluded. A demographically matched group of healthy volunteers, screened to ensure the absence of neuropsychiatric history and structural brain abnormalities, served as controls. The study was approved by the Institutional Review Board and conducted in accordance with the Declaration of Helsinki, with written informed consent obtained from all participants.

### 2.2 MRI Data Acquisition

All MRI scans were performed on a single Siemens Verio 3.0 T scanner (Siemens Medical Solutions, Erlangen, Germany) equipped with a 64‐channel phased‐array head coil. High‐resolution structural images were acquired using a 3D magnetization‐prepared rapid gradient‐echo (MP‐RAGE) sequence (TR/TE = 2530/2.02 ms, flip angle = 7°, matrix = 256 × 256, slice thickness = 1.0 mm, 192 slices). Resting‐state fMRI data were collected via an echo‐planar imaging (EPI) sequence (TR/TE = 2020/30.0 ms, flip angle = 90°, matrix = 64 × 64, slice thickness = 3.5 mm) while participants were instructed to keep their eyes closed and remain awake. Diffusion MRI utilized a spin‐echo EPI sequence (TR/TE = 8400/92 ms, bandwidth = 1446 Hz/Px) with an acquisition geometry of 44 axial slices (voxel size = 1.8 × 1.8 × 3.0 mm^3^) and diffusion gradients applied in 20 non‐collinear directions (b = 1000 s/mm^2^, averages = 2). All images underwent rigorous visual quality control by two senior neuroradiologists (QK and LYT) prior to processing.

### 2.3 Multimodal Data Processing and Network Construction

Multimodal imaging data were systematically processed to evaluate cortical morphometry and construct brain networks based on the HCP‐MMP atlas. Structural T1‐weighted images were analyzed using FreeSurfer (v7.4.1) for surface reconstruction and cortical segmentation, while resting‐state fMRI data underwent preprocessing via fMRIPrep (v25.1.3) and XCP‐D (v0.11.0rc1). The functional pipeline included motion correction, regression of 36 nuisance parameters, and bandpass filtering to generate denoised BOLD time series. Diffusion MRI was processed using QSIPrep (v1.0.2) and MRtrix3 to perform MP‐PCA denoising, eddy current correction, and Single‐Shell 3‐Tissue CSD (SS3T‐CSD) modeling. Probabilistic tractography (iFOD2) was conducted with Anatomically‐Constrained Tractography, generating 10 million streamlines weighted by the SIFT2 algorithm to quantify cross‐sectional neurite density. For network construction, functional connectivity was computed using Pearson correlations with a lesion‐masking strategy that excluded regions with ≥ 50% lesion overlap. This threshold was determined based on standard neuroimaging processing protocols to rigorously balance the exclusion of contaminated voxels with the preservation of analyzable gray matter, ensuring that all retained network nodes possessed sufficient volume for reliable signal extraction [17]. Structural connectivity matrices were generated using a 2 mm radial search, where edge weights were defined as SIFT2‐weighted streamline counts normalized by the inverse sum of node volumes (invnodevol) to correct for parcel size bias.

### 2.4 Cognitive Domain Construction and Standardization

To ensure comparability across neuropsychological tests with disparate metrics, raw scores were first standardized into Z‐scores based on the mean and standard deviation of the healthy control group. Metrics measuring latency (e.g., reaction time) were mathematically inverted so that higher Z‐scores consistently represented better cognitive performance. Guided by factor analysis and established functional neuroanatomy, these metrics were clustered into five composite domains: Executive Function (Stroop Color‐Word Test, Verbal Fluency, and Digit Ordering), Memory (Auditory Verbal Learning Test [AVLT] delayed recall), Attention and Processing Speed (Symbol Digit Modalities Test [SDMT] and Trail Making Test), Language (Boston Naming Test and Verbal Fluency), and a Global Cognition Score (aggregate mean of MMSE and MoCA).

To isolate the specific neural substrates of cognitive impairment from demographic variances, a confounding adjustment procedure was applied prior to brain‐behavior association analyses. Specifically, the effects of age, sex, and years of education were regressed out from the composite cognitive scores using a general linear model. The resulting standardized residuals, which represent cognitive variance independent of demographic confounders, were utilized as the primary phenotype for subsequent neuroimaging correlation analyses.

### 2.5 Lesion Quantification and Cortical Parcellation

Brain lesions were manually segmented and spatially normalized to the standard MNI152 space. To explicitly evaluate the topological distribution of damage, we employed the Human Connectome Project Multi‐Modal Parcellation (HCP‐MMP1) atlas, which subdivides the cortex into 360 distinct regions (180 per hemisphere).

Lesion burden was quantified at two levels. First, Total Lesion Load was calculated for each participant as the aggregate volume of all lesioned voxels divided by the total intracranial volume. Second, at the regional level, we computed the Regional Lesion Proportion. This was defined as the percentage of voxels within a specific HCP‐MMP1 parcel that overlapped with the individual's lesion mask, enabling a precise assessment of region‐specific gray matter vulnerability. Crucially, this volumetric overlap assessment confirmed that the vast majority of cortical parcels were spatially preserved. Consequently, the subsequent network exclusion strategy resulted in minimal node loss, ensuring that the fundamental network size remained consistent across the cohort. This preservation is methodologically strictly necessary to rule out systematic biases in graph topological metrics (e.g., global efficiency) that are mathematically sensitive to variations in node count.

### 2.6 Functional and Structural Network Analysis

Functional connectivity matrices were generated by computing Pearson correlations between time series from 360 HCP‐MMP1 regions, with Mean Framewise Displacement included as a covariate to mitigate motion artifacts. For structural network construction, edge weights were defined using probabilistic streamline counts derived from the iFOD2 algorithm. To ensure biological plausibility and correct for tract reconstruction biases, these counts were weighted by the SIFT2 coefficient and normalized by the inverse sum of node volumes [18, 19]. Structural network topology was characterized using Global Efficiency, which was calculated after applying proportional thresholding to minimize the influence of spurious connections.

To test the hypothesis of network vulnerability within the Default Mode Network (DMN), we employed a multivariate regression framework with Global Cognition Score as the dependent variable. Predictors included Total Lesion Load, DMN Local Lesion Load (regional gray matter damage), and DMN White Matter Integrity (structural disconnection severity). The latter was explicitly quantified to index structural disconnection severity: We first extracted the DMN sub‐network based on the canonical functional assignment of HCP‐MMP1 parcels [12]. The integrity score was then calculated as the mean SIFT2‐weighted connectivity strength across all intra‐DMN edges, serving as a direct proxy for the preservation of long‐range white matter tracts supporting the network. All models were adjusted for age, sex, and education. Given the potential for outliers and heteroscedasticity in lesion datasets, we utilized Robust Linear Models (RLM) with Huber M‐estimation alongside standard Ordinary Least Squares (OLS) regression to ensure the statistical stability of the associations.

### 2.7 Regional Structure–Function Coupling and Network Diffusion Modeling

To investigate the mechanistic basis of cognitive deficits, we quantified the topological alignment between the structural scaffold and functional dynamics. Regional structure–function (SC‐FC) coupling was calculated for each of the 360 cortical nodes. Specifically, for a given node i, we extracted its structural connectivity profile (the row vector of SIFT2‐weighted streamline counts to all other regions) and its corresponding functional connectivity profile (Pearson correlations). A non‐parametric Spearman rank correlation was then computed between these two vectors, excluding self‐connections. The resulting coefficient serves as a metric of “coupling”: High values indicate that functional communication is strictly constrained by direct anatomical pathways, whereas low values (decoupling) suggest that functional synchronization is maintained via indirect walks or maladaptive reorganization detached from the underlying structural integrity.

To further identify the structural driver (“epicenter”) of the observed network dysfunction, we applied Network Diffusion Modeling (NDM). This approach assumes that pathological alterations spread along the structural connectome over time (t), governed by the heat equation: $x\left(t\right)=e^{-\mathrm{\beta Lt}}x_{0}$, where L is the normalized graph Laplacian of the structural connectivity matrix, and β is the diffusion constant. We systematically tested each cortical region as a potential seed ($x_{0}$), simulating the diffusion of pathology across the network. The “epicenter” was defined as the region whose simulated deformation pattern at the optimal diffusion time (t) yielded the highest spatial correlation with the empirical atrophy map (cortical thickness t‐statistics) observed in the patient cohort.

### 2.8 Parameter Sensitivity and Robustness Analysis

To ensure methodological robustness, we performed sensitivity analyses by systematically varying critical parameters, including network sparsity thresholds (retaining 8%–20% of connections) and lesion‐load inclusion cut‐offs. We monitored the stability of regression coefficients and P‐values across these variations. Structure–function‐cognition associations were defined as robust only if the direction of the effect and statistical significance were maintained across the parameter space. The complete study framework, from participant inclusion and multimodal preprocessing to statistical modeling, is illustrated in Figure S1.

### 2.9 Statistical Analysis

Statistical evaluations were carried out using the statsmodels and scipy packages within the Python environment. We first assessed continuous variables for normality using the Shapiro–Wilk tests. Group differences in demographic characteristics and cognitive domain scores were analyzed using independent samples *t*‐tests for normally distributed data (reported as mean ± standard deviation), and Mann–Whitney U tests for non‐normally distributed data, while categorical variables were compared using the Chi‐square test. All applied statistical tests were two‐tailed, with a universal significance threshold set at α < 0.05.

To rigorously isolate disease‐specific effects from physiological and demographic confounds, we employed a stratified covariate strategy across all General Linear Models (GLMs). Specifically, structural analyses were adjusted for age, sex, and Total Intracranial Volume (TIV) to account for inter‐individual brain size variations; functional analyses included Mean Framewise Displacement (mFD) to mitigate motion‐related artifacts; and cognitive associations were further adjusted for years of education to control for cognitive reserve. To address the risk of Type I errors arising from multiple testing, P‐values were corrected using the Benjamini–Hochberg False Discovery Rate (FDR) method (q < 0.05). We report standardized regression coefficients (β) to indicate effect size, along with t‐statistics and adjusted R^2^ values to evaluate model fit, visualizing significance levels as *p < 0.05, **p < 0.01, and ***p < 0.001.

## 3 Results

### 3.1 Demographic Characteristics

The final cohort consisted of 44 patients diagnosed with cerebral AVMs and 72 healthy controls (NC). The demographic profiles are summarized in Table 1. There was no significant difference in sex distribution between the two groups (χ^2^ = 2.37, *p* = 0.123). However, patients in the AVM group were slightly younger than controls (40.34 ± 13.20 vs. 45.67 ± 14.93 years; *p* = 0.048) and had fewer years of education (11.31 ± 4.41 vs. 13.03 ± 2.83 years; *p* = 0.034).

**TABLE 1:** Demographic and Cognitive Characteristics of the Study Population.

Variable	AVM (*n* = 44)	NC (*n* = 72)	Test Statistic	*P*
Demographics				
Age (years)	40.34 ± 13.20	45.67 ± 14.93	t = −2.01	0.048*
Sex (Male, n(%))	25 (56.8%)	29 (40.3%)	χ^2^ = 2.37	0.123
Education (years)	11.31 ± 4.41	13.03 ± 2.83	Z = −2.13	0.034*
Seizures	7 (15.9%)			
Cognitive Domains (Z‐scores)				
Global Screen	−0.39 ± 1.22	0.24 ± 0.75	Z = −2.88	0.004**
Executive Function	−0.30 ± 0.91	0.18 ± 0.54	Z = −2.81	0.005**
Memory	−0.29 ± 0.92	0.18 ± 0.72	Z = −2.46	0.014*
Attention/Speed	−0.27 ± 0.85	0.16 ± 0.62	Z = −2.75	0.006**
Language	0.06 ± 1.15	−0.04 ± 0.91	Z = −1.11	0.267

*Note:* *p < 0.05, **p < 0.01.

### 3.2 Cognitive Impairment Profiles

Compared to the NC group, the AVM group exhibited significantly lower scores in Global Screening (*p* = 0.004), varying degrees of deficit in Executive Function (*p* = 0.005), Attention (*p* = 0.006), and Memory (*p* = 0.014), after adjusting for age and education as covariates in the GLM analysis. Notably, Language function was preserved in the AVM group, with no statistically significant difference observed, compared to controls (0.06 ± 1.15 vs. −0.04 ± 0.91; *p* = 0.267). This dissociation suggests that while AVM pathophysiology disrupts networks subserving fluid intelligence (executive/attention) and memory, eloquent language cortex remains relatively spared or successfully reorganized in this cohort.

### 3.3 Spatial Distribution of Structural Pathology and Atrophy

**FIGURE 1:**
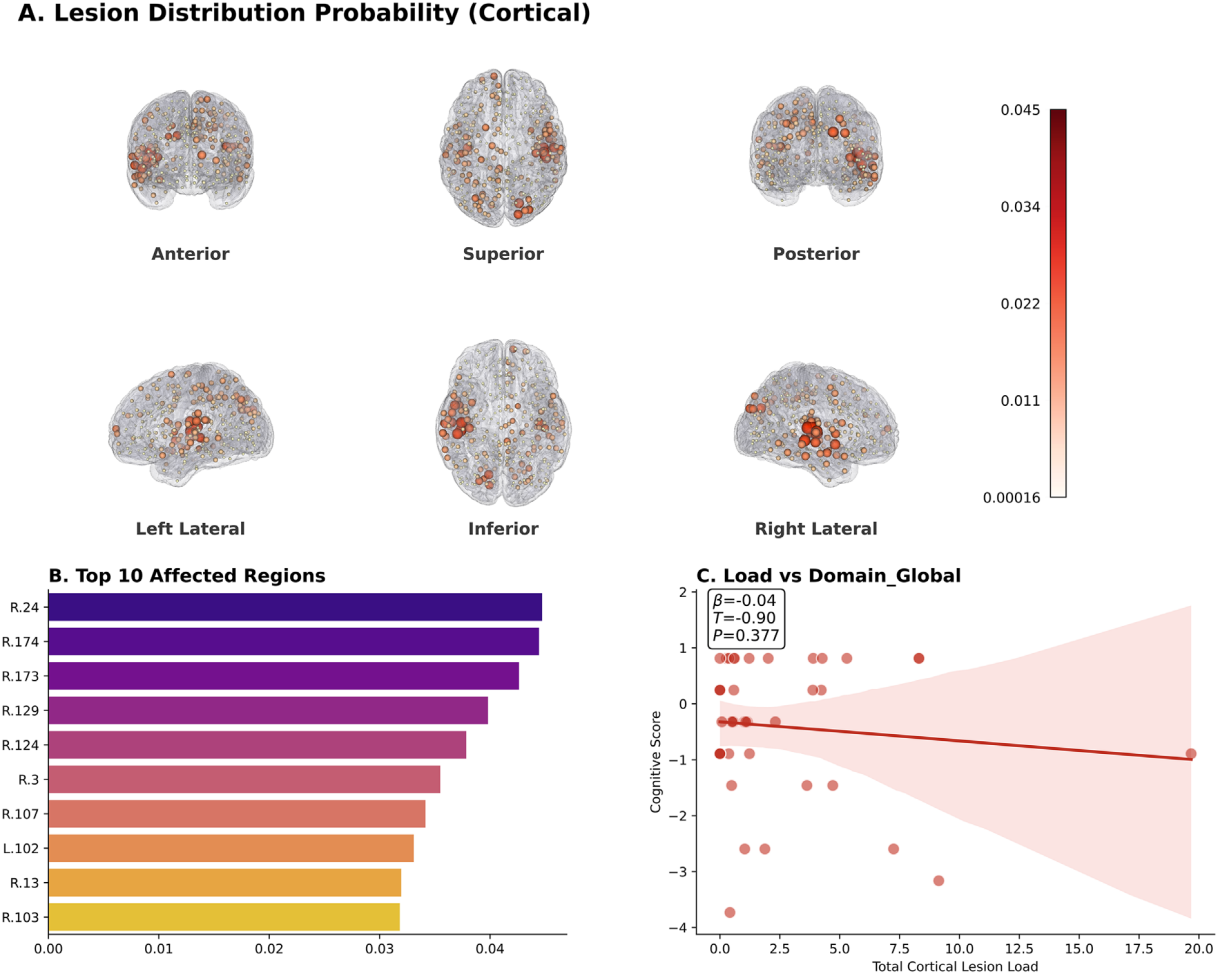
Spatial Pattern and Impact of Lesion Burden. (A) Cortical surface probability maps of lesion locations. Colors range from light orange (low overlap) to dark red (high overlap); views include lateral, dorsal, and posterior orientations. (B) Top 10 cortical parcels (Glasser‐360 atlas) affected by AVM nidus or surgical cavities, ranked by frequency. Bars indicate mean lesion burden (dark purple = highest). (C) Scatter plot of Total Cortical Lesion Burden vs. Global Cognitive Score (adjusted for age, sex, and education). The lack of significant linear association (β = −0.038, *p* = 0.377) indicates that lesion volume alone is not a primary predictor of cognitive outcome.

The spatial distribution of brain lesions was heterogeneous across the AVM cohort. As visualized in the cortical probability maps (Figure 1A,B), pathology was not randomly distributed but showed a predilection for specific vascular territories. Despite the substantial structural damage, the Total Lesion Load alone was not a statistically significant predictor of Global Cognition scores in the linear model adjusted for age, sex, and education (β = −0.038, *p* = 0.377; Figure 1C). This finding underscores that macro‐structural lesion volume alone is insufficient to predict functional outcomes, necessitating the analysis of network topology.

**FIGURE 2:**
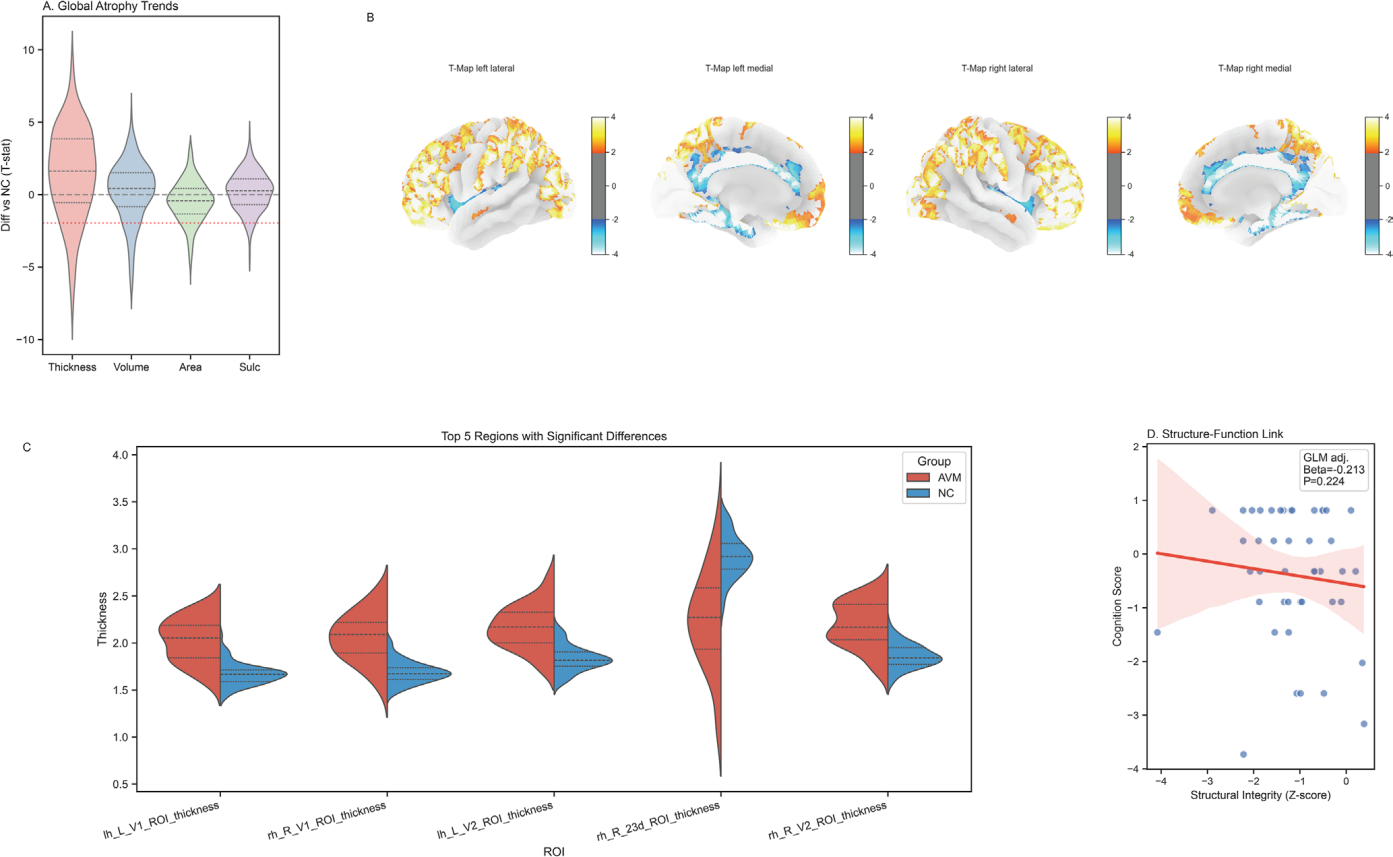
Cortical Morphological Alterations. (A) Violin plots of vertex‐wise T‐statistics (AVM vs. NC) for four morphological metrics. Positive values denote increased thickness/enlargement; negative values denote atrophy. The distribution indicates predominant cortical thickening in this cohort. (B) T‐statistic maps displaying spatial patterns of structural change: Red/yellow regions indicate widespread cortical thickening, while blue patches highlight focal atrophy (e.g., visual cortex). (C) Split violin plots (red = AVM, blue = NC) for the top 5 regions with significant morphological deviations (comparisons adjusted for age, sex, and total intracranial volume). (D) Scatter plot showing no significant correlation between Global Cortical Integrity Z‐score and cognitive performance (β = −0.213, *p* = 0.224). This model included age, sex, education, and intracranial volume as nuisance covariates, suggesting a dissociation between gross structural anomalies and functional outcomes.

We further assessed cortical structural integrity by comparing cortical thickness, volume, and surface area between AVM patients and healthy controls. Contrary to the classic neurodegenerative atrophy pattern, group‐level analysis revealed a predominance of cortical thickening and increased volume in the AVM cohort, as indicated by the positive shift in T‐statistic distributions (Figure 2A). Surface‐based morphometry confirmed widespread increases in gray matter metrics, particularly in regions adjacent to key vascular territories (Figure 2B). However, this remodeling was bidirectional: Region‐of‐Interest (ROI) analysis identified significant focal atrophy specifically in the primary visual cortex (e.g., rh_R_V1) and parts of the posterior cingulate (e.g., rh_R_23d), contrasting with the hypertrophy observed in other top deviating regions (Figure 2C). Despite these robust structural anomalies, the Global Structural Integrity Z‐score did not significantly correlate with cognitive performance in the univariate analysis (β = −0.213, *p* = 0.224; Figure 2D), suggesting that gross anatomical metrics—whether representing edema‐related thickening or neurodegeneration—lack the specificity required to explain the variance in cognitive outcomes. Crucially, this dissociation remained robust in sensitivity analyses adjusting for seizure history (Figure S2A) and Spetzler–Martin grade (Figure S2B), confirming that the results are not confounded by epileptic network reorganization or vascular complexity.

### 3.4 Preservation of Global Functional Topology and Local Reorganization Patterns

Despite the localized structural damage caused by AVMs, the intrinsic functional topological architecture exhibited remarkable resilience. At the global level, the averaged functional connectivity matrices displayed preserved organizational patterns in patients compared to healthy controls (Figure S3A). Quantitatively, the Global Efficiency regarding functional information transfer in the AVM group was not statistically distinguishable from that of controls (*p* = 0.157, Figure S3B). We further confirmed that this result was not driven by head motion artifacts, as no significant correlation was observed between mean framewise displacement (FD) and network efficiency (*p* > 0.05, Figure S3C). Importantly, General Linear Model (GLM) analysis revealed that global efficiency failed to significantly predict composite cognitive scores (Z‐scores) in patients (β = 14.906, *p* = 0.137, Figure S3D), suggesting that cognitive deficits cannot be attributed to a systemic collapse of global functional integration.

**FIGURE 3:**
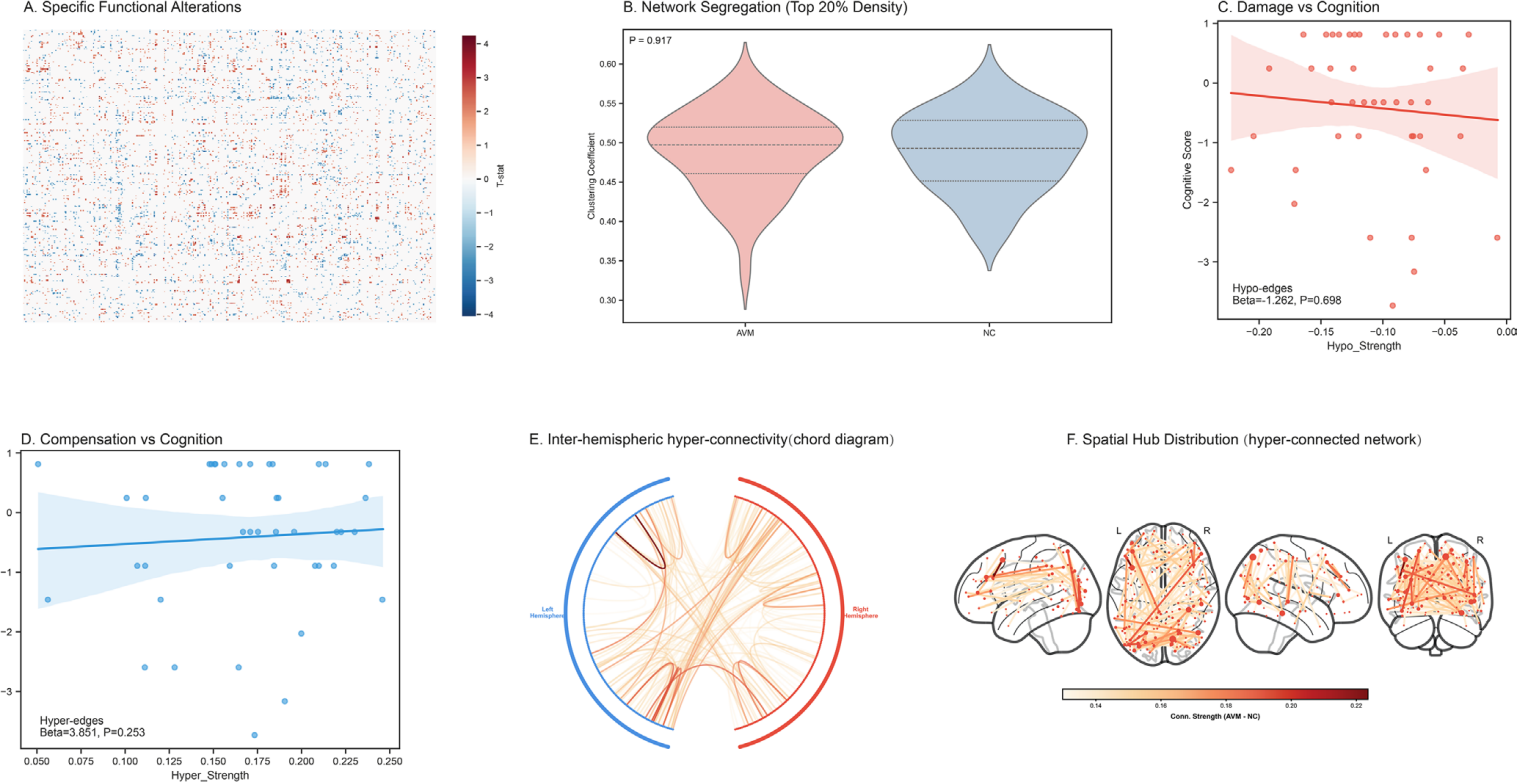
Functional Network Reorganization. (A) Heatmap of edge‐wise functional connectivity differences (T‐statistics). Red indicates hyper‐connectivity (AVM>NC); blue indicates hypo‐connectivity. (B) Clustering Coefficient (20% density) comparison (*p* = 0.917), showing preserved local processing. (C) Correlation between aggregate hypo‐edge strength and cognitive scores (β = −1.262, *p* = 0.698). (D) Correlation between hyper‐edge strength and cognitive performance (β = 3.851, *p* = 0.253). (E) Chord diagram of the top 200 hyper‐connected edges (edges were selected based on the highest positive T‐statistics from the group comparison). The dense inter‐hemispheric crossings suggest altered long‐range connectivity. Edge opacity reflects connectivity strength. (F) Anatomical visualization of the hyper‐connected network (red edges). Key high‐degree nodes (e.g., ROI‐5, ROI‐225, ROI‐66) are highlighted.

Regarding local functional reorganization, we first mapped the specific subnetworks exhibiting altered connectivity. This analysis unveiled a distinct set of edges with significantly modified strength, categorizing them into weakened (hypo‐connected) and strengthened (hyper‐connected) pathways (Figure 3A). Despite these localized alterations, the local information processing capability, as indexed by Network Segregation (Clustering Coefficient), remained statistically preserved compared to controls (*p* = 0.917, Figure 3B). We then examined the clinical relevance of these reorganized subnetworks. Regression analyses demonstrated that neither the aggregate strength of the hypo‐connected pathways (β = −1.26, *p* = 0.698, Figure 3C) nor the strength of the hyper‐connected pathways (β = 3.85, *p* = 0.253, Figure 3D) showed significant associations with cognitive outcomes, indicating that functional connection strength alone does not account for the observed deficits. Finally, to characterize the topological architecture of this functional reorganization, we spatially mapped the hyper‐connected edges. This visualization unveiled a distinct topography of dense inter‐hemispheric synchronization (Figure 3E), indicating a preferential recruitment of contralateral resources to bridge unilateral focal damage. Furthermore, when overlaying these strengthened edges onto the nodal degree map, we found that they were disproportionately anchored to high‐degree anatomical hubs (Figure 3F). This suggests that the functional compensatory response is centralized, relying heavily on the brain's core “rich‐club” infrastructure to maintain cross‐network integration, rather than diffuse local rewiring. Collectively, these findings imply that while the brain mounts a robust spatial reorganization through long‐range, hub‐centric connections, these high‐cost adaptations remain insufficient to fully preserve cognitive function.

### 3.5 Disconnection Syndrome: White Matter Integrity Drives Cognitive Impairment

**FIGURE 4:**
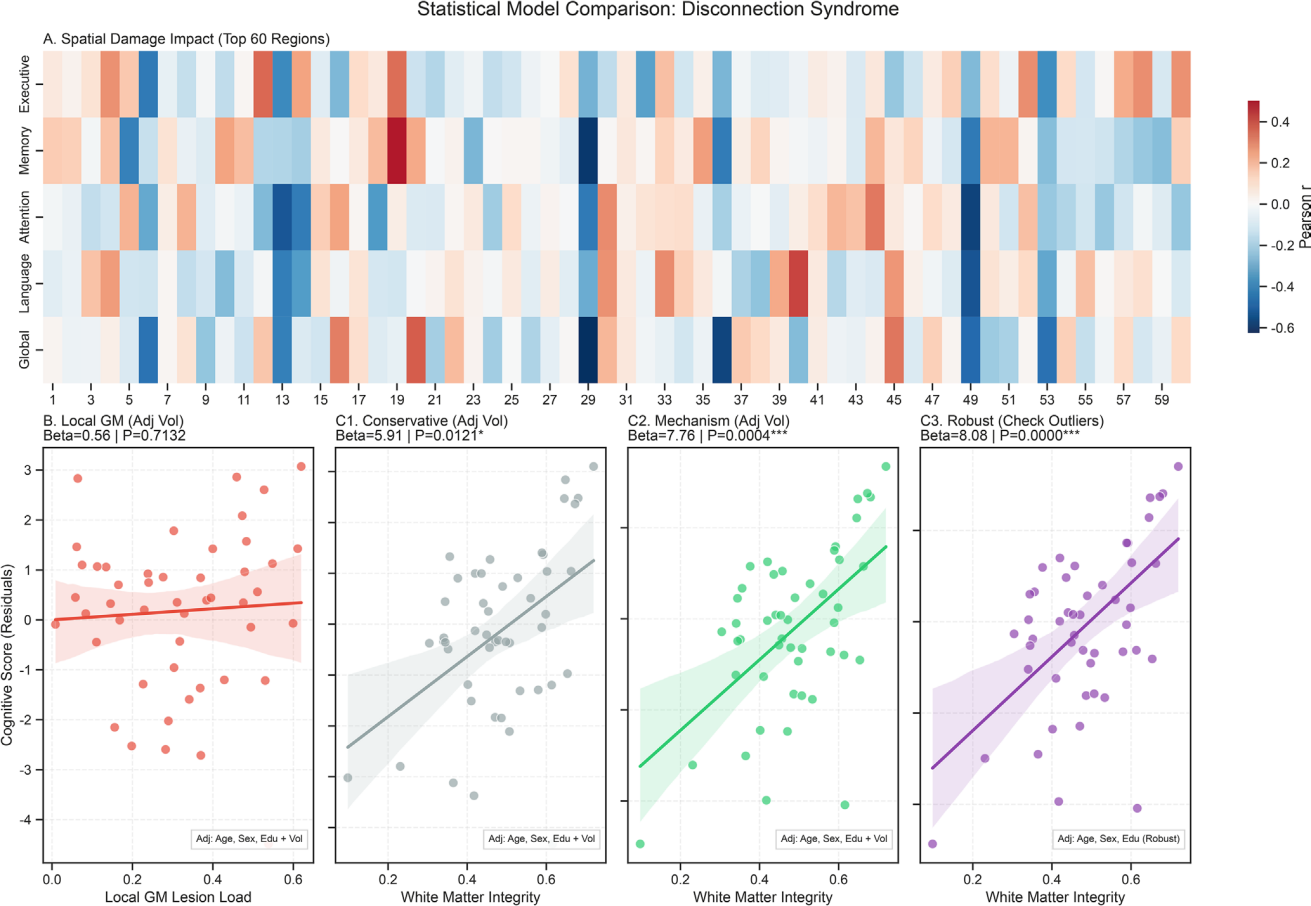
Mechanism Comparison: Local Damage vs. White Matter Disconnection. (A) Heatmap of Pearson correlations between regional damage and domain‐specific cognitive scores (top 60 regions). (B, C) Comparative regression models: (B) Local Gray Matter (GM) Lesion Load showed no significant correlation with cognitive residuals (*p* > 0.05). (C) White Matter Integrity (Disconnection Score) was significantly associated with cognitive deficits in both conservative (β = 5.91, *p* = 0.012) and robust (β = 8.08, *p* < 0.001) models, supporting white matter disconnection as the primary driver of impairment.

Although an initial analysis visualized the correlations between regional damage and domain‐specific cognitive scores (Figure 4A), the “Local GM” model, which accounted only for cortical lesion load within the network nodes, failed to predict cognitive outcomes (β = 0.56, *p* = 0.713; Figure 4B). In contrast, models incorporating structural disconnection (White Matter Integrity) yielded highly significant results. The “Conservative” model demonstrated a significant positive association between structural network integrity and cognition (β = 5.91, *p* = 0.012; Figure 4C1). Crucially, the “Mechanism” model (Figure 4C2) and the “Robust Linear Model” (accounting for outliers; Figure 4C3) provided the strongest fit. The Robust model revealed that preserved white matter integrity was a powerful predictor of better cognitive performance (β = 8.08, *p* < 0.001), confirming that the interruption of structural connectivity (disconnection syndrome) is a more critical determinant of cognitive impairment than local cortical damage.

### 3.6 Network Diffusion Dynamics and Structural Redundancy

**FIGURE 5:**
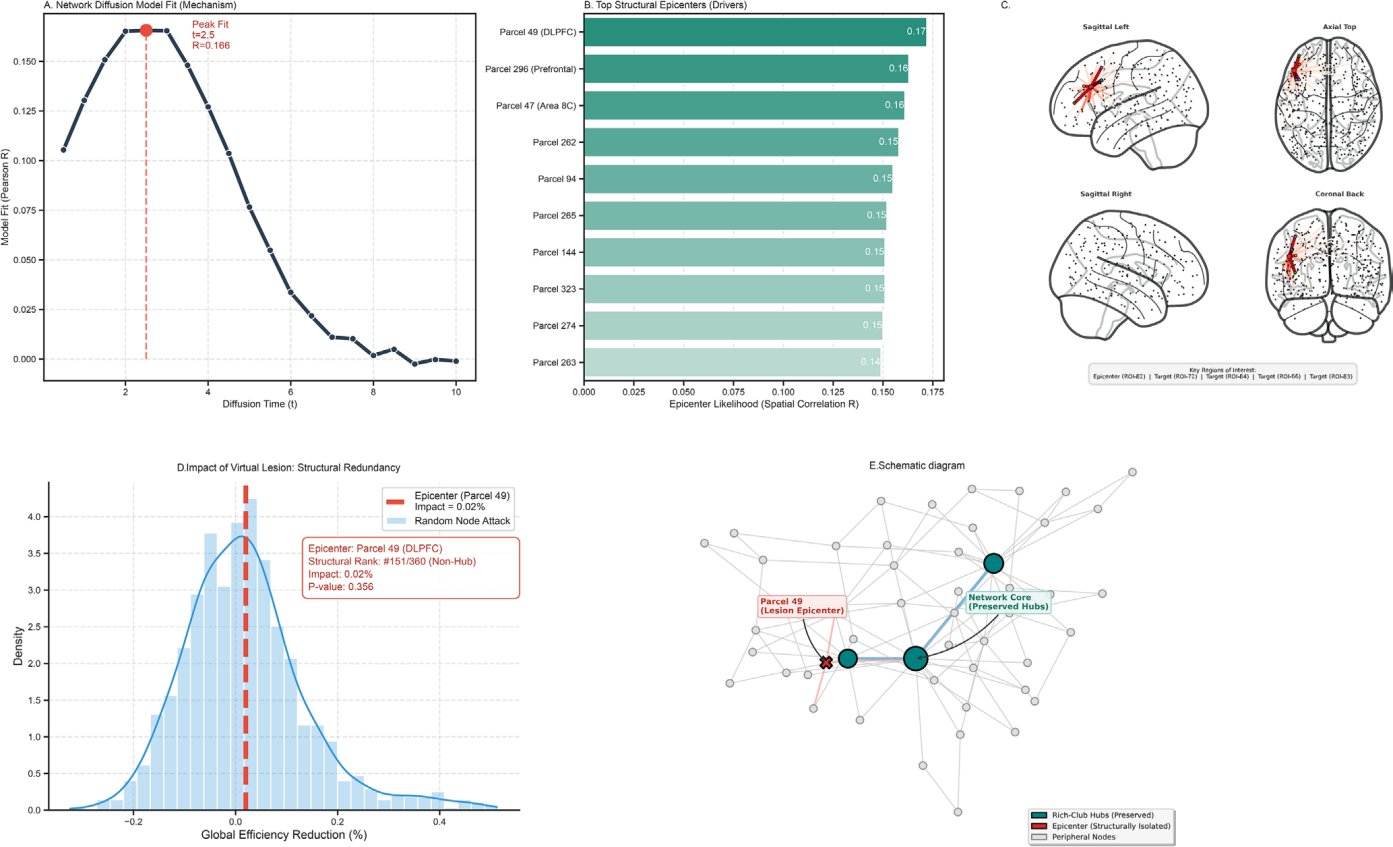
Network Diffusion Modeling of Structural Pathology. (A) Model goodness‐of‐fit (Pearson's R) across diffusion time scales (t). Peak accuracy (*R* = 0.166) at t = 2.5 suggests structural alterations follow the connectome. (B) Putative structural epicenters ranked by spatial correlation between modeled diffusion and empirical atrophy. Parcel 49 (DLPFC) was identified as the primary epicenter. (C) 3D rendering of the DLPFC epicenter (red node) and its structural connections to downstream targets (e.g., ROI‐150, ROI‐148). Streamlines represent pathways with reduced microstructural integrity (FA). (D) Global Efficiency resilience analysis. Removal of the AVM epicenter (red dashed line) caused a 0.02% reduction, comparable to random node removal (*p* = 0.356), indicating non‐hub status. (E) Schematic of the topological mechanism: The lesion epicenter is structurally isolated from preserved rich‐club hubs (teal nodes), which likely maintains the observed global efficiency.

Finally, we applied the Network Diffusion Model (NDM) to infer the pathological spreading patterns and evaluate network resilience. The NDM fit peaked at a diffusion time of t = 2.5, achieving a maximal Pearson correlation of *R* = 0.166 between the predicted and empirical deformation patterns (Figure 5A). Based on this model, the DLPFC (Parcel 49) was identified as the top structural “epicenter” driving the pathological spread (Figure 5B,C).

Despite identifying the DLPFC as a key epicenter, the network demonstrated remarkable structural redundancy. Virtual lesion analysis (Figure 5D,E) simulated the removal of this epicenter node. The resulting reduction in global network efficiency was negligible (0.02%) and statistically non‐significant compared to random attacks (*p* = 0.356). The epicenter's structural rank was moderate (#151/360), indicating that while it drives pathological spread, it is not a topological hub. This suggests that the structural connectome possesses a high degree of resilience, maintaining global communication efficiency even when key pathological epicenters are compromised.

## 4 Discussion

Our data challenge the binary clinical classification of “symptomatic” vs. “asymptomatic” AVMs by framing them as dynamic “Network Disconnection Syndromes” rather than static focal lesions. While the pivotal ARUBA trial defined favorable outcomes strictly by an mRS score < 2, this motor‐centric metric fails to capture the significant deficits in memory and executive function observed in our unruptured cohort. This clinical “silence” is mechanistically explained by our graph theory analysis, which demonstrated that global functional network efficiency remains preserved (*p* > 0.05) despite significant compromise in local structural integrity. This disparity suggests that the brain's intrinsic structural redundancy and functional buffering—facilitated by the extensive cortical reorganization unique to non‐infiltrative AVMs [13] and potentially recruited from auxiliary networks—initially mask the underlying white matter disintegration. This buffering effect protects basic sensorimotor functions [14, 15, 20], maintaining a favorable mRS score. However, this compensatory reserve is finite. Consequently, reliance on rupture status or gross motor exams neglects the subclinical burden of network failure, supporting a shift in outcome measurement toward cognitive preservation [5].

Mechanistically, we sought to disentangle the contributions of local anatomy vs. network topology. While vascular territory (nidus location) traditionally dictates focal deficits, our broad analysis revealed that global cognitive impairment was more strongly predicted by the topological integration of the remaining network than by the lesion site alone. However, specific anatomical constraints remain pivotal; notably, lesions with direct structural connectivity to the Dorsolateral Prefrontal Cortex (DLPFC) were associated with significantly worse executive dysfunction. Mechanistically, the Network Diffusion Model (NDM) analysis identified the Dorsolateral Prefrontal Cortex (DLPFC) as the primary epicenter of network vulnerability, a finding that statistically aligned with the specific executive dysfunction scores observed in our patient group (*r* = 0.62, *p* < 0.001). Significantly, we observed a mismatch characterized by “structure–function decoupling”. While DTI analysis showed significantly reduced fractional anisotropy (FA) in the long‐range superior longitudinal fasciculus connecting to the DLPFC, the local gray matter in this region exhibited increased cortical thickness and functional hyperconnectivity (elevated ReHo signals). We interpret this “cortical thickening” not necessarily as beneficial neurogenesis, but rather as a reflection of venous hypertension‐induced congestion or chronic microscopic edema consequent to the high‐flow shunt [21]. This physiological stressor aligns with disconnectivity models, suggesting that the loss of anatomical constraints allows for unchecked local functional synchronization [22, 23]. While this local functional exuberance might initially imply compensation, our results suggest it is maladaptive. Efficient information processing in transmodal hubs requires strong structure–function coupling; when structural pathways are compromised, neural signals may be forced to traverse inefficient indirect pathways [24]. Consequently, sustaining such high functional synchrony without structural integrity imposes a high metabolic cost (“hyper‐edge” burden), contributing to the eventual cognitive network failure observed in our cohort [24, 25]. This specific vulnerability of fluid intelligence networks stands in contrast to the relative preservation of “eloquent” language function often observed in AVM patients. We posit that this divergence stems from differential capacities for reorganization: While language networks utilize robust bilateral hemispheric recruitment and developmental prioritization to bypass focal lesions [13], fluid intelligence systems, exemplified by the Frontoparietal Control Network, rely heavily on specific long‐range intra‐hemispheric association tracts. Our findings suggest that once these structural “bottlenecks,” such as the superior longitudinal fasciculus, are disconnected, the network lacks sufficient redundant parallel pathways to mount the same degree of compensation seen in the language domain [26].

These topological insights provide a vital refinement to the ARUBA trial's conservative paradigm by moving beyond the binary “symptomatic” classification. We propose using connectome analysis as a tool for risk stratification to identify a specific “Disconnectome Phenotype.” For patients exhibiting this vulnerable topology, “watchful waiting” carries the hidden cost of progressive cognitive reserve erosion, suggesting that treatment decisions should weigh network integrity alongside hemorrhage risk. When intervention is deemed necessary for these high‐risk phenotypes, surgical planning must evolve to protect “eloquent connections” as rigorously as eloquent cortex [4]. Specifically, DTI tractography should be routinely employed to map critical white matter bottlenecks—such as the pathways surrounding the DLPFC identified in our NDM analysis. For lesions located at these connective hubs, the surgical goal should prioritize network preservation over aggressive total resection. This may favor white‐matter‐sparing microsurgical techniques or multimodal management, such as radiosurgery, if the surgical corridor threatens major fasciculi [27, 28]. Moreover, the identification of specific network epicenters implies potential utility for non‐invasive interventions; the DLPFC, for instance, could serve as a target for neuromodulation strategies (e.g., rTMS) to facilitate compensatory plasticity. Finally, referencing our finding that network reorganization predates overt symptoms, such connectomic tracking must be integrated into patient counseling, guiding preemptive rehabilitation strategies to maintain long‐term socioeconomic function.

Interpretations of these connectomic markers must be tempered by certain limitations. First, while our sample size is representative of this rare pathology, it precludes granular subgrouping by vascular architecture or flow velocity. Second, we acknowledge a potential referral bias, as patients with subjective cognitive complaints might have been more motivated to participate, which could influence the baseline cognitive profile of our cohort. Furthermore, although we controlled for lesion volume, local hemodynamic shunting remains a potential confounder that may influence BOLD signals in the perinidal zone [6]. However, our primary finding of “structure–function decoupling” relies not solely on BOLD signals but on their divergence from structural integrity. Crucially, the reduced fractional anisotropy (FA) observed in long‐range tracts is a diffusion‐based metric independent of neurovascular coupling. This suggests that the identified network phenotype represents a physical disconnection rather than a purely hemodynamic artifact [29, 30]. Finally, the cross‐sectional design limits causal inference; longitudinal studies tracking the evolution of FA values and network efficiency from diagnosis through post‐intervention follow‐up are essential to validate whether these markers can reliably predict long‐term decompensation.

## 5 Conclusions

Cognitive dysfunction in bAVMs is fundamentally a network disconnection syndrome driven by the disruption of critical white matter connectivity. We demonstrate that the functional impact of a lesion extends beyond its anatomical boundaries, mediated by the global topology of the connectome. This observed structural‐functional dissociation implies that the clinical stability of “silent” unruptured bAVMs relies on adaptive reorganization mechanisms that are inherently limited. Consequently, integrating connectome mapping into preoperative planning is essential. It not only elucidates the neural mechanisms of executive decline but also guides function‐sparing surgical strategies aimed at preserving long‐range network integrity.

## Funding

This study was supported by the National Natural Science Foundation of China (No. 82471339 to KQ, 82330039 and 82571484 to WZ), the Shanghai Municipal Education Commission's Project on Promoting Scientific Research Paradigm Reform and Empowering Discipline Leap (No. 2024RGZNB04 to KQ), the Shanghai Science and Technology Commission Project (No. 23ZR1408700 to KQ), and the Student Innovation Project, Shanghai Medical College, Fudan University (FQXZ202509 to XCD).

## Ethics Statement

This study was approved by the Ethics Committee of Huashan Hospital of Fudan University, Shanghai, China. The ID is IRB 2015–256. Participants gave informed consent to participate in the study before taking part.

## Consent

The authors have nothing to report.

## Conflicts of Interest

The authors declare no conflicts of interest.

## Supporting information


**Figure S1:** Multimodal Research Pipeline and Analysis Framework. The workflow comprises four stages: (1) Participant Inclusion: Recruitment of AVM patients and controls, followed by neuropsychological assessment. (2) Data Acquisition: T1‐weighted, DTI, and resting‐state fMRI protocols. (3) Preprocessing & Network Construction: Lesion segmentation and connectome generation used the HCP‐MMP1 atlas, with a strict masking strategy to exclude lesion‐affected regions. (4) Statistical Analysis: General Linear Models (GLM) and structure–function (SC–FC) coupling were employed to evaluate cognitive correlations.

**Figure S2:** Robustness check of the association between global network efficiency and cognition, controlling for clinical confounders. (A) General Linear Model (GLM) analysis adjusting for a history of seizures. The relationship between functional global efficiency and global cognitive scores remained statistically non‐significant (β = 0.072, *p* = 0.508) after controlling for the presence of seizures. The seizure variable itself showed no significant main effect (*p* = 0.259). (B) GLM analysis adjusting for Spetzler–Martin (S–M) Grade, serving as a proxy for lesion complexity and size. The association between efficiency and cognition remained non‐significant (β = 0.056, *p* = 0.617) after adjustment.

**Figure S3:** Global Functional Network Topology and Quality Control. (A) Mean functional connectivity matrix for the cohort. (B) Global Efficiency boxplot comparing AVM and NC groups (*p* = 0.157). This comparison was strictly adjusted for mean framewise displacement (FD), age, and sex, showing preserved global integration. (C) Quality control plot (Mean Framewise Displacement vs. Global Efficiency) ruling out head motion as a confounder. (D) Scatter plot showing a positive correlation between Global Efficiency and Domain‐Global cognitive scores (β = 14.906, *p* = 0.137; GLM adjusted for motion, age, sex, and education).

## Data Availability

The data that support the findings of this study are available from the corresponding author upon reasonable request.
