# Supplementary material for: Network Disconnection Syndrome in Unruptured Brain Arteriovenous Malformations: A Multimodal Connectome Study
Source: CNS Neurosci Ther. 2026 Mar 11;32(3):e70819. doi: 10.1002/cns.70819 (PMC12977985; doi:10.1002/cns.70819)
Supplement: Supplementary file 1 — Figure S1: Multimodal Research Pipeline and Analysis Framework. The workflow comprises four stages: (1) Participant Inclusion: Recruitment of AVM patients and controls, followed by neuropsychological assessment. (2) Data Acquisition: T1‐weighted, DTI, and resting‐state fMRI protocols. (3) Preprocessing & Network Construction: Lesion segmentation and connectome generation used the HCP‐MMP1 atlas, with a strict masking strategy to exclude lesion‐affected regions. (4) Statistical Analysis: General Linear Models (GLM) and structure–function (SC–FC) coupling were employed to evaluate cognitive correlations. [file CNS-32-e70819-s003.pdf]

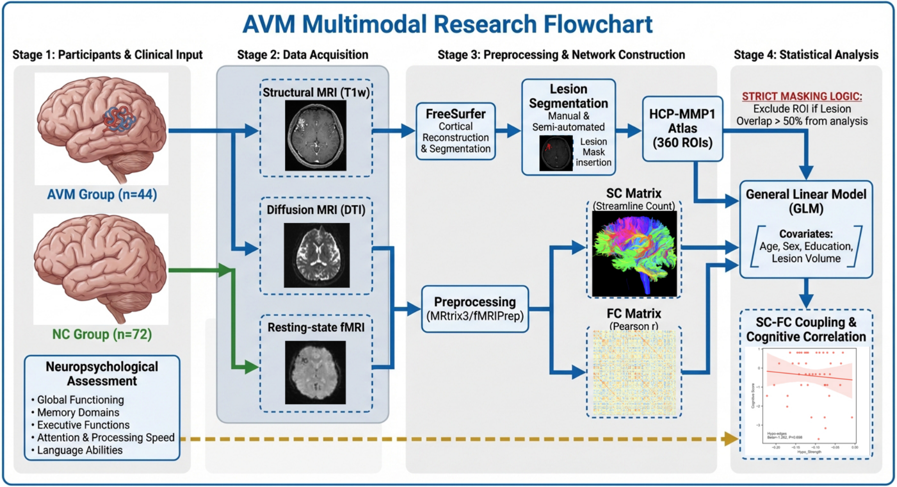
AVM Multimodal Research Flowchart
Stage 1: Participants & Clinical Input
Stage 2: Data Acquisition
Stage 3: Preprocessing & Network Construction
Stage 4: Statistical Analysis
AVM Group (n=44)
NC Group (n=72)
Neuropsychological Assessment
• Global Functioning
• Memory Domains
• Executive Functions
• Attention & Processing Speed
• Language Abilities
Structural MRI (T1w)
Diffusion MRI (DTI)
Resting-state fMRI
FreeSurfer
Cortical Reconstruction & Segmentation
Lesion Segmentation
Manual & Semi-automated
Lesion Mask insertion
HCP-MMP1 Atlas (360 ROIs)
Preprocessing (MRtrix3/fMRIPrep)
SC Matrix
(Streamline Count)
FC Matrix
(Pearson r)
STRICT MASKING LOGIC:
Exclude ROI if Lesion Overlap > 50% from analysis
General Linear Model (GLM)
Covariates:
Age, Sex, Education, Lesion Volume
SC-FC Coupling & Cognitive Correlation
