# Supplementary material for: Network Disconnection Syndrome in Unruptured Brain Arteriovenous Malformations: A Multimodal Connectome Study
Source: CNS Neurosci Ther. 2026 Mar 11;32(3):e70819. doi: 10.1002/cns.70819 (PMC12977985; doi:10.1002/cns.70819)
Supplement: Supplementary file 2 — Figure S2: Robustness check of the association between global network efficiency and cognition, controlling for clinical confounders. (A) General Linear Model (GLM) analysis adjusting for a history of seizures. The relationship between functional global efficiency and global cognitive scores remained statistically non‐significant (β = 0.072, p = 0.508) after controlling for the presence of seizures. The seizure variable itself showed no significant main effect (p = 0.259). (B) GLM analysis adjusting for Spetzler–Martin (S–M) Grade, serving as a proxy for lesion complexity and size. The association between efficiency and cognition remained non‐significant (β = 0.056, p = 0.617) after adjustment. [file CNS-32-e70819-s001.pdf]

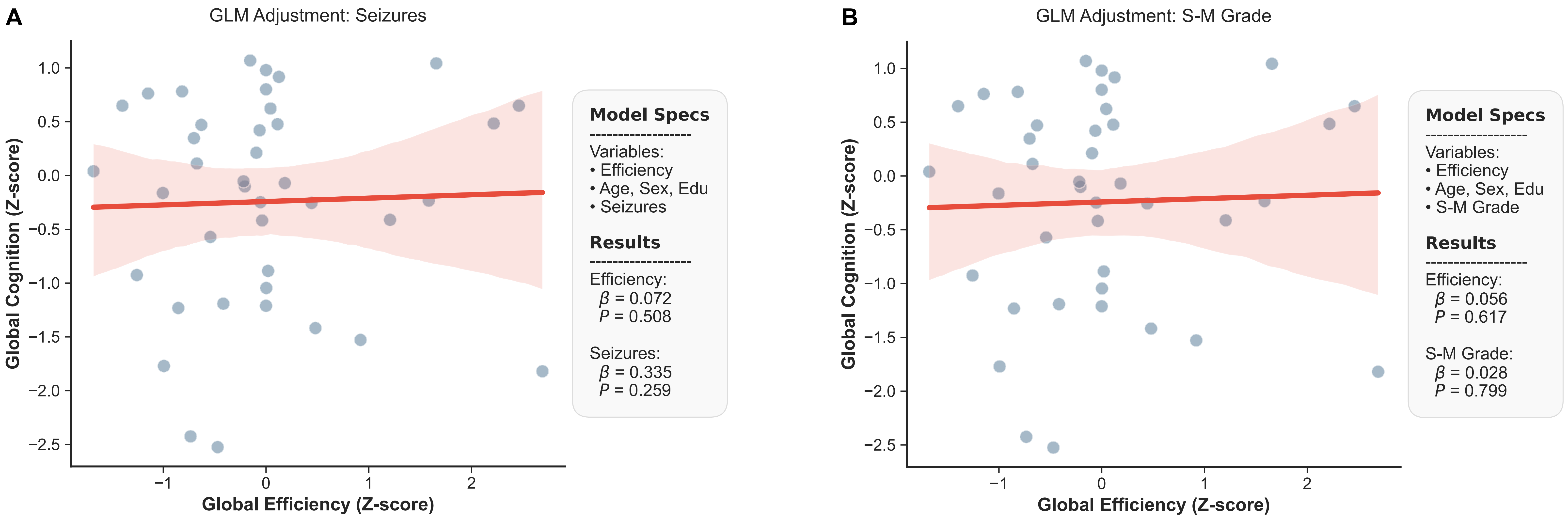

A
GLM Adjustment: Seizures
Global Cognition (Z-score)
Global Efficiency (Z-score)
1.0
0.5
0.0
−0.5
−1.0
−1.5
−2.0
−2.5
−1
0
1
2
Model Specs
Variables:
• Efficiency
• Age, Sex, Edu
• Seizures
Results
Efficiency:
β = 0.072
P = 0.508
Seizures:
β = 0.335
P = 0.259
B
GLM Adjustment: S-M Grade
Global Cognition (Z-score)
Global Efficiency (Z-score)
Model Specs
Variables:
• Efficiency
• Age, Sex, Edu
• S-M Grade
Results
Efficiency:
β = 0.056
P = 0.617
S-M Grade:
β = 0.028
P = 0.799
