# Supplementary material for: Network Disconnection Syndrome in Unruptured Brain Arteriovenous Malformations: A Multimodal Connectome Study
Source: CNS Neurosci Ther. 2026 Mar 11;32(3):e70819. doi: 10.1002/cns.70819 (PMC12977985; doi:10.1002/cns.70819)
Supplement: Supplementary file 3 — Figure S3: Global Functional Network Topology and Quality Control. (A) Mean functional connectivity matrix for the cohort. (B) Global Efficiency boxplot comparing AVM and NC groups (p = 0.157). This comparison was strictly adjusted for mean framewise displacement (FD), age, and sex, showing preserved global integration. (C) Quality control plot (Mean Framewise Displacement vs. Global Efficiency) ruling out head motion as a confounder. (D) Scatter plot showing a positive correlation between Global Efficiency and Domain‐Global cognitive scores (β = 14.906, p = 0.137; GLM adjusted for motion, age, sex, and education). [file CNS-32-e70819-s002.pdf]

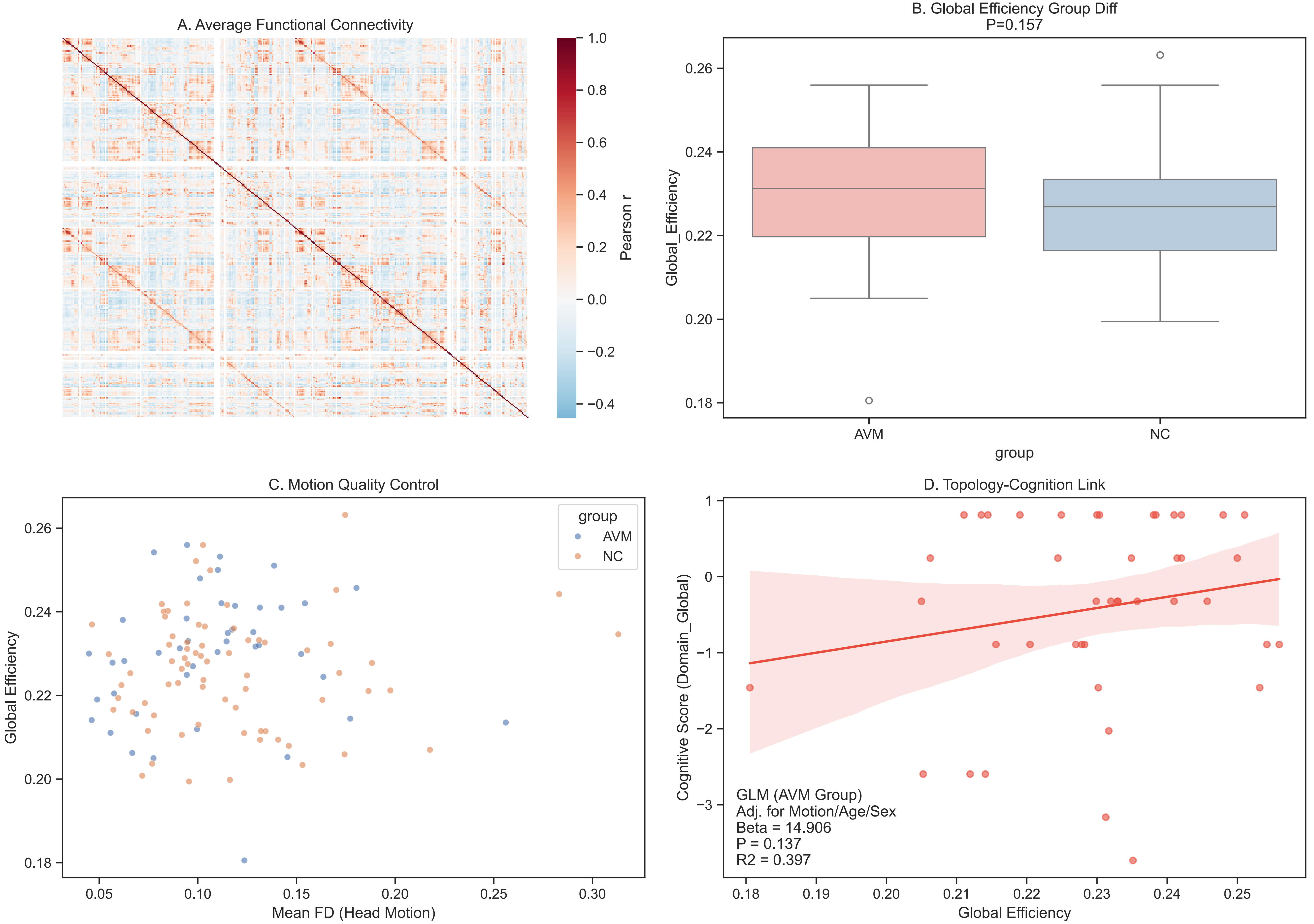

A. Average Functional Connectivity
Pearson r
1.0
0.8
0.6
0.4
0.2
0.0
−0.2
−0.4
B. Global Efficiency Group Diff
P=0.157
Global_Efficiency
0.26
0.24
0.22
0.20
0.18
AVM
NC
group
C. Motion Quality Control
group
AVM
NC
Global Efficiency
Mean FD (Head Motion)
0.05
0.10
0.15
0.20
0.25
0.30
D. Topology-Cognition Link
Cognitive Score (Domain_Global)
1
0
−1
−2
−3
GLM (AVM Group)
Adj. for Motion/Age/Sex
Beta = 14.906
P = 0.137
R2 = 0.397
0.18
0.19
0.20
0.21
0.22
0.23
0.24
0.25
Global Efficiency
